# On the possible role of stimulation duration for after-effects of transcranial alternating current stimulation

**DOI:** 10.3389/fncel.2015.00311

**Published:** 2015-08-10

**Authors:** Daniel Strüber, Stefan Rach, Toralf Neuling, Christoph S. Herrmann

**Affiliations:** ^1^Experimental Psychology Laboratory, Department of Psychology, Center for Excellence ‘Hearing4all’, European Medical School, Carl von Ossietzky UniversityOldenburg, Germany; ^2^Research Center Neurosensory Science, University of OldenburgOldenburg, Germany; ^3^Department of Epidemiological Methods and Etiologic Research, Leibniz Institute for Prevention Research and Epidemiology – BIPSBremen, Germany; ^4^Center for Mind/Brain Sciences (CIMeC), University of TrentoRovereto, Italy

**Keywords:** EEG, electroencephalography, tACS, NIBS, non-invasive brain stimulation, entrainment, synaptic plasticity

## Abstract

Transcranial alternating current stimulation is a novel method that allows application of sinusoidal currents to modulate brain oscillations and cognitive processes. Studies in humans have demonstrated transcranial alternating current stimulation (tACS) after-effects following stimulation durations in the range of minutes. However, such after-effects are absent in animal studies using much shorter stimulation protocols in the range of seconds. Thus, stimulation duration might be a critical parameter for after-effects to occur. To test this hypothesis, we repeated a recent human tACS experiment with a short duration. We applied alpha tACS intermittently for 1 s duration while keeping other parameters identical. The results demonstrate that this very short intermittent protocol did not produce after-effects on amplitude or phase of the electroencephalogram. Since synaptic plasticity has been suggested as a possible mechanism for after-effects, our results indicate that a stimulation duration of 1 s is too short to induce synaptic plasticity. Future studies in animals are required that use extended stimulation durations to reveal the neuronal underpinnings. A better understanding of the mechanisms of tACS after-effects is crucial for potential clinical applications.

## Introduction

Non-invasive brain stimulation (NIBS) has become a powerful tool in neuroscience. Recently, a number of NIBS methods have been developed that are able to modulate brain oscillations due to entrainment comprising transcranial alternating current stimulation (tACS) and oscillatory transcranial direct current stimulation (otDCS; Herrmann et al., 2013). On the one hand, tACS is able to modulate electroencephalography (EEG) oscillations during the time of stimulation. For example, 10 Hz tACS shifted the frequency of subjects’ EEG alpha rhythm towards 10 Hz and enhanced its amplitude (Helfrich et al., 2014b). This demonstrates that tACS is able to entrain an endogenous brain oscillation which is important in order to influence cognitive processes during the time of stimulation. On the other hand, it is important to know whether tACS also results in after-effects that outlast the end of stimulation—a pre-requisite for its clinical application in disorders involving disturbed brain oscillations, i.e., dynamic diseases such as schizophrenia or ADHD (Herrmann and Demiralp, 2005; Uhlhaas and Singer, 2006). Therefore, it has been investigated whether tACS and otDCS result in after-effects on physiology, cognition, or motor processes (see Table 1).

**Table 1:** **Examples of human tACS studies demonstrating after-effects**.

Article	Stimulation frequency	Stimulation intensity	Stimulation duration	Stimulated area/tACS electrodes	Type of after-effect	Duration of after-effect
Angelakis et al. (2013)	15 Hz	20 min	1.5 mA	C3, C4	Therapeutic (pain)	30 days
Bergmann et al. (2009)	0.8 Hz	30 min intermittent	1.5 mA	M1	Motor cortex	5 min
Chaieb et al. (2011)	1,2,5 kHz	10 min	1 mA	M1	Motor cortex excitability	30–60 min
Garside et al. (2015)	0.75 Hz	25 min intermittent	0.55 mA	F3, F4	EEG (disruption of low frequency power)	24 min
Groppa et al. (2010)	0.8 Hz	10 min	1.5 mA	M1	Motor cortex excitability	20 min 20 min
Helfrich et al. (2014a)	10 Hz	20 min	1 mA	Oz, Cz	EEG alpha power	1 min
Helfrich et al. (2014b)	40 Hz	20 min	1 mA	MT	EEG gamma coherence	20 min
Moliadze et al. (2010)	140 Hz	10 min	1 mA	M1	Motor cortex excitability	60 min
Neuling et al. (2013)	8–12 Hz	20 min	1.5 mA	Cz, Oz	EEG alpha power	30 min
Schutter and Hortensius (2011)	5/20 Hz	10 min	1 mA	C3, C4	Motor cortex excitability	5 min
Strüber et al. (2014)	40 Hz	15 min	1.023 ± 0.62 mA	P7-PO7, P8-PO8	EEG gamma coherence coherence	3 min
Vossen et al. (2015)	IAF	12 min intermittent	1.35–2.0 mA	PO7-PO9, PO8-PO10	EEG alpha power	2 min
Wach et al. (2013a)	10 Hz	10 min	1 mA	M1	Motor performance	30 min
Wach et al. (2013b)	10 Hz	10 min	1 mA	M1	MEG gamma corticomuscular coherence	30–38 min
Zaehle et al. (2010)	IAF	10 min	1.12 ± 0.49 mA	PO9, PO10	EEG alpha power	3 min

*Note that the durations of the after-effects of some studies were inferred indirectly from the study design. Furthermore, the durations do not reflect the maximum possible range of after-effects since no study so far assessed the complete time course of tACS after-effects from start to finish. This table is not intended to be exhaustive. Note: M1, primary motor cortex; MT, a 4 × 1 electrode montage over medial temporal cortex; electrode positions refer to the extended 10–10 system*.

With respect to motor processes, 5 min of 10 Hz tACS over the primary motor cortex led to an improvement of the acquisition and early consolidation during implicit motor learning in a serial reaction time task (Antal et al., 2008). After-effects were also reported for the excitability of motor cortex as indexed via motor potentials evoked by transcranial magnetic stimulation for at least 20 min after the end of stimulation (Groppa et al., 2010).

Regarding physiology, tACS at a frequency in the human EEG alpha range (8–12 Hz) is able to enhance the amplitude of the alpha rhythm (Zaehle et al., 2010; Neuling et al., 2012a). This after-effect lasts at least for 30 min (Neuling et al., 2013). In addition, Strüber et al. (2014) were able to demonstrate that tACS at 40 Hz enhanced inter-hemispheric phase synchronization which in turn alters visual perception. The enhanced phase synchronization between hemispheres (coherence) persists for at least 3 min after the end of stimulation. In a follow-up study with similar perceptual effects, it has been shown that the inter-hemispheric coherence modulation outlasts stimulation offset by approximately 20 min (Helfrich et al., 2014a).

However, the neural mechanisms underlying tACS and its after-effects are not well understood yet. Animal experiments offer an ideal way to investigate both the immediate on-line effects during stimulation as well as the off-line effects after the end of stimulation. Recently, the physiological mechanisms that underlie the observed tACS effects have been revealed via intracranial recordings in ferrets (Fröhlich and McCormick, 2010). The authors stimulated ferrets intracranially and simultaneously recorded local field potentials (LFPs) and multiunit activity (MUA). Cortical slices were stimulated *in vitro* and multi-unit activity was recorded simultaneously revealing that weak sinusoidal voltages elicit neuronal spikes. The spiking activity synchronized to different driving frequencies, suggesting that neuronal firing can be entrained to the electrically applied field. From that study, however, it was not clear whether weak currents also penetrate the skull and still have similar effects upon neuronal activity. Another group has addressed this question by stimulating rats with electrodes on the surface of the skull while recording neural activity intracranially (Ozen et al., 2010). These authors were able to show that extracranially applied sinusoidal currents can result in similar effects. Whereas intracranial recordings in animals were able to shed light onto the immediate effects during stimulation, typically no after-effects are observed (Deans et al., 2007). In a recent review, Reato et al. (2013: p. 6) stated: “Yet, it is important to note that none of the animal studies reviewed above report lasting effects, i.e., as soon as the AC fields are turned off, the observed effects seemingly disappear”.

However, we would like to stress that these animal studies did not aim at assessing after-effects specifically. Thus, long-lasting tACS effects were not to be expected and the differences between human and animal studies regarding long-lasting tACS effects do not reflect an inconsistency of findings. Rather, these differences demonstrate the necessity to specify common parameters for investigating the neuronal underpinnings of tACS after-effects as a basis for clinical tACS applications. When comparing human and animal stimulation protocols, it becomes evident that several important stimulation parameters like duration, intensity, frequency and electrode montage differ between experiments (Herrmann et al., 2013; Reato et al., 2013). Since these parameters might modulate tACS after-effects interactively, it is important to study them in isolation. Here, we focus on stimulation duration as a main factor since animal experiments typically applied stimulation durations in the range of seconds, whereas human experiments usually apply the currents in the range of minutes. Therefore, it is of great relevance to study the effect of brief stimulation duration as typical for animal experiments on after-effects in humans, and—*vice versa*—to study longer stimulation durations as typical for human experiments in animals. As a starting point, here we have repeated a previous human tACS experiment that found after-effects with 20 min stimulation duration (Neuling et al., 2013) but applied shorter durations of 1 s. We applied tACS in the alpha range intermittently for 1 s while keeping other parameters like intensity and electrode montage identical. Since we were interested in the effects of single one-second trains, the interval between the tACS trains varied randomly to avoid phase-synchrony between consecutive tACS trains.

## Materials and Methods

### Participants

Thirteen healthy adults (seven females, mean age 22.5 ± 3.1 years) took part in this experiment and received monetary compensation for their participation. All participants were right handed and had normal or corrected-to-normal vision. They had no history of psychiatric or neurological diseases and were under no current medication affecting the central nervous system. Participants were informed about all aspects of the study and gave their written informed consent prior to the experiment. The study protocol was designed and performed according to the declaration of Helsinki and was approved by the local ethics committee of the Carl von Ossietzky Universität, Oldenburg.

### Experimental Procedure

**Figure 1:**
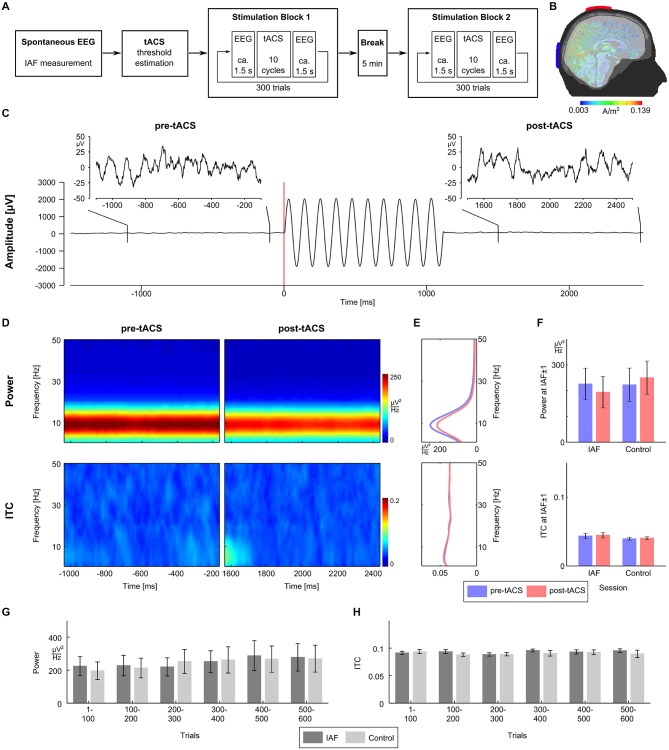
**Experimental procedure and results. (A)** The experiment consisted of two sessions recorded on two separated days. Sessions started with 3 min of spontaneous EEG recordings to estimate the individual alpha frequency (IAF), before the thresholds for skin sensation and phosphene perception were measured. Afterwards participants completed two stimulation blocks with 300 trials each separated by a 5 min break. **(B)** tACS electrodes were centered over Cz and Oz of the 10/20 system. A finite-element model simulation revealed that this montage results in current densities that are highest in the posterior cortex (see Neuling et al., 2012b for details). **(C)** Exemplary EEG data (electrode P3) from a typical trial. The participant was stimulated with ten cycles of tACS at 9 Hz starting at 0 ms. For each trial, the pre-tACS epoch from −1100 to −100 ms relative to tACS onset and post-tACS epoch from 1500 to 2500 ms relative to tACS onset were chosen for analysis. **(D)** Time-frequency plots of power (upper row) and intertrial coherence (ITC, bottom row) for the pre-tACS (left column) and the post-tACS epoch (right column) in the IAF session. **(E)** Power (upper panel) and ITC (lower panel) averaged across the pre-tACS (blue) and the post-tACS epoch (red) in the IAF session. **(F)** Mean power (upper panel) and mean ITC (lower panel) at IAF ± 1 Hz do not differ significantly between the pre-tACS (blue) and the post-tACS epoch (red), neither in the IAF session, nor in the control session. **(G)** Time course of mean power (left) at IAF ± 1 Hz in steps of 100 trials does not show differences between the IAF session (dark gray) and the control session (light gray). **(H)** Time course of mean ITC (right) at IAF ± 1 Hz in steps of 100 trials also shows no differences between the IAF session (dark gray) and the control session (light gray).

Participants completed two identical sessions (duration 1 h; see Figure 1A) on two separate days: the IAF-session with stimulation at their individual alpha frequency (IAF), and the control-session with stimulation at a control frequency of IAF*3.1 Hz (order of sessions balanced across participants). Three minutes of spontaneous EEG were recorded while participants had their eyes closed to determine their IAFs (mean IAFs, IAF session: 9.7 Hz ± 1.03 Hz; control session: 9.7 ± 0.9 Hz). Afterwards, participants were familiarized with the tACS-induced skin sensations or visual phosphenes and their individual stimulation intensity was determined in a threshold estimation procedure. Then, the two experimental blocks consisting of 300 tACS trials were conducted, separated by a break of 5 min. Each trial consisted of 1.5 s of resting EEG, followed by approximately 1 s of tACS (IAF-session: exactly 10 cycles at the IAF; control-session: between 26 and 40 cycles at the control frequency), 1.5 s of resting EEG afterwards, and a random inter-trial duration between one and 3 s. To ensure wakefulness and attention, participants had to complete a visual detection task throughout the whole session where they had to respond to infrequently presented lights by pressing a button.

### Electrical Stimulation

tACS was applied via two conductive rubber electrodes (5 × 7 cm^2^, NeuroConn, Ilmenau, Germany) attached over Cz and Oz location underneath the EEG recording cap (Figure 1B). Stimulation electrode positions were chosen in order to affect the occipital cortex (Neuling et al., 2012b). Impedances were kept below 10 kΩ with Ten20 conductive paste (Weaver and company, Aurora, Colorado). A sinusoidally alternating current was applied at IAF using a battery-driven stimulator (DC-Stimulator Plus, NeuroConn, Ilmenau, Germany). To determine individual stimulation intensities, the thresholds for skin sensations and phosphenes were determined for all participants after the spontaneous EEG block. Using the method of limits, tACS at IAF was applied with an initial intensity of 1.5 mA (peak-to-peak) for 1 s and the intensity was either increased or decreased in steps of 0.1 mA depending on whether or not the participant reported a sensation. The individual stimulation intensity was chosen to be 0.100 mA below the highest intensity at which the stimulation was not noticed by the participant (mean stimulation intensities, IAF session: 0.758 mA ± 0.301 mA; control session: 0.877 mA ± 0.386 mA).

### EEG

EEG was recorded from 25 sintered Ag-AgCl electrodes mounted in an elastic cap (Easycap, Falk Minow, Munich, Germany) with a standard 10–20 system layout, using a nose-tip reference and ground electrode at Fpz. Electrode impedances were kept below 10 kΩ. Signals were recorded using Brain Vision Recorder (Brain Products GmbH, Gilching, Germany) with a sampling rate of 5000 Hz in the range of ± 3.2768 mV at a resolution of 0.1 μV. EEG data were digitally stored on hard disk for offline analysis.

### Data Analysis

EEG data were analyzed using MATLAB R2012a (The MathWorks Inc., Natick, MA, USA) and EEGLAB 13.0.4.3b (Delorme and Makeig, 2004). For the determination of the IAF, the data from the spontaneous EEG block were epoched into segments of 1 s duration. A fast Fourier transformation (FFT) was performed on the first 100 artifact-free segments and the resulting spectra were averaged. The power peak in the alpha range (8–12 Hz) was considered as IAF and used as stimulation frequency. For each trial of the experimental block, data were epoched from −1100 to −100 ms relative to tACS onset for the pre-tACS epoch, and from 1500 to 2500 ms relative to tACS onset for the post-tACS epoch (Figure 1C). A trial was rejected when either of the pre-tACS or post-tACS epochs contained unique, non-stereotyped artifacts. ICA was used to remove artifact activity related to eye blinks, electrocardiographic artifacts, as well as other sources of non-cerebral activity (Jung et al., 2000) and data were sampled down to 250 Hz afterwards. Electrodes P3 and P4 were chosen for data analysis. Oscillatory power and inter-trial coherence (ITC) were calculated for the pre-tACS and the post-tACS epochs by means of FFT using the EEGLAB function *newtimef*. To evaluate the evolution both measures over the course of a session, power and ITC were calculated for six bins of 100 consecutive trials each.

## Results

To investigate whether intermittent tACS does influence oscillatory power, we compared power between the pre-tACS and the post-tACS epochs. We expected that entrainment effects due to tACS would manifest as an increase in power. However, neither time frequency power plots of both epochs (Figure 1D, upper panel), nor mean power spectra across epochs (Figure 1E, upper panel) revealed evidence for a power increase. Mean power at IAF ± 1 Hz was entered into a 2 × 2 repeated measurements ANOVA with factors session (IAF vs. control) and epoch (pre vs. post) but did not reveal any significant main effects or interactions (all *p* > 0.27; Figure 1F, upper panel).

Since an entrainment effect induced by tACS could also lead to a higher degree of phase alignment across trials in the post-tACS epoch as compared to the pre-tACS epoch, the same analyses were repeated for ITC. Again, neither time frequency plots of both epochs (Figure 1D, lower panel), nor mean ITC across epochs (Figure 1E, lower panel) showed significant differences. Mean ITC at IAF ± 1 Hz was entered into a 2 × 2 repeated measurements ANOVA with factors session and epoch, but also did not reveal any significant main effects or interactions (all *p* > 0.37; Figure 1F, lower panel).

Although no changes in oscillatory power or phase alignment could be detected when comparing pre-tACS and post-tACS epochs, the intermittent stimulation regime could still lead to detectable changes when applied over the course of 1 h. To investigate this possibility, repeated measurement ANOVAs with factors session (IAF vs. control) and time (6 bins of 100 trials each) were calculated for power (Figure 1G) and ITC (Figure 1H). For oscillatory power, the AVOVA revealed a significant main effect for time (*F*_(5,60)_ = 4.8, *p* < 0.002) indicating a power change over the course of a session. This effect, however, could not be attributed to the tACS at IAF since no significant effects were observed for session (*F*_(1,12)_ = 2.8; *p* = 0.12) or the interaction between session and time (*F*_(5,60)_ = 1.8; *p* = 0.12). Thus, both the IAF and control group showed an increase of alpha activity over time probably reflecting fatigue. For ITC, the ANOVA did not reveal any significant effects for factors session and time, or the interaction between session and time (all *p* > 0.25).

## Discussion

Our results show that with such a short intermittent protocol (1 s stimulation), no after-effects occurred with respect to EEG amplitude or phase. Together with our previous findings of pronounced after-effects following 20 min of stimulation (Neuling et al., 2013), this study provides evidence for a role of stimulation duration in creating tACS after-effects. What might be the underlying mechanisms?

In human tACS research, it has been a matter of debate whether after-effects are due to entrainment of brain oscillations or neural plasticity or a combination thereof. In principle, an entrained brain oscillation can remain at an elevated amplitude after the end of the entraining stimulation but typically decays after a few cycles (Halbleib et al., 2012). Already in the first experiment that showed enhanced EEG alpha amplitudes after the end of a tACS stimulation at IAF it has been argued that most probably synaptic plasticity was the reason for this enhancement (Zaehle et al., 2010). The authors had used a neural simulation incorporating spike-timing dependent plasticity in order to support their argumentation. Recently, another experiment supported this notion (Vossen et al., 2015). The authors have used intermittent intervals of tACS stimulation at IAF and pauses. Stimulation intervals could either be in phase to previous stimulation intervals (continuous condition) or out of phase (discontinuous condition). If after-effects were due to entrainment, only continuous stimulation should be effective. However, the observed after-effect of enhanced EEG alpha amplitude did not depend upon continuous as compared to discontinuous stimulation. In addition, the study by Vossen et al. (2015) only showed after-effects for their intermittent protocols if 8 s stimulation duration was used but not for 3 s stimulation duration. Taken together, these findings were interpreted as evidence for synaptic plasticity but not entrainment being responsible for the observed after-effects. Nevertheless, the authors argued that entrainment during stimulation might be required for subsequent effects of synaptic plasticity.

However, Chaieb et al. (2011) found that tACS in the range of kHz also resulted in neural plasticity of the motor system lasting for 30–60 min after stimulation. Since no brain oscillations are known in this high-frequency range, this finding argues against entrainment being required during tACS stimulation for subsequent plasticity. In addition to temporal constraints, Groppa et al. (2010) showed that plastic changes of motor cortex excitability were dependent also on the intensity of tACS stimulation. The authors found plastic changes for a stimulation intensity of 1.5 mA but not for 0.75 mA. The induced changes of motor cortex excitability lasted for 20 min.

Interestingly, 40 Hz tACS can lead to plastic changes of physiological parameters even in the absence of power effects. Strüber et al. (2014) observed a pure coherence effect in the gamma range without a corresponding power increase. In a subsequent study, 40 Hz tACS resulted in enhanced inter-hemispheric gamma-band coherence that lasted for 20 min after the end of stimulation, again without a corresponding power effect (Helfrich et al., 2014a). Intriguingly, the observed effects on gamma coherence in both studies were not constrained to the stimulation frequency of 40 Hz but rather spanned a wide frequency region from about 30 to 60–100 Hz. According to Miller et al. (2009) such wide-band phenomena represent spiking activity of large neural populations rather than neural oscillations which would result in sharp peaks in a frequency spectrum. This is in line with the fact that 40 Hz tACS did not enhance EEG power in the two abovementioned studies which would have required an ongoing oscillation. Thus, the enhanced gamma coherence between hemispheres most probably reflects an increase in the synchronization of neural spikes rather than an increase of spike rate.

Taken together, current human tACS studies suggest that synaptic plasticity and not entrainment is the decisive factor in producing after-effects. Thus, stimulation durations need to be long enough to induce plastic changes within neural networks. According to our results, brief tACS trains of 1 s duration are not sufficient. Future studies in animals and humans are required that use a spectrum of stimulation durations to determine how this stimulation parameter determines the origin and duration of after-effects. We strongly emphasize the need for additional animal research using longer stimulation durations to elucidate the underlying mechanisms of tACS after-effects at the cellular and network level. Especially, *in vivo* studies with extended stimulation durations would be critical for a direct comparison with human studies. As already stated by Reato et al. (2013: p. 6): “[…] long-term effects at the cellular level must mediate the long-term effects observed in human studies, thus, there is an urgent need to clarify the underlying mechanisms”. In addition, a better understanding of the mechanisms that underlie the tACS after-effects is essential for effective clinical applications of tACS (Fröhlich et al., 2015). Future experiments on the effects of tACS on cognition and behavior should not only assess the immediate effects during stimulation but also the duration of after-effects and how they might depend on stimulation parameters such as intensity, duration and the sequence of stimulation and non-stimulation intervals (intermittent protocols).

## Conflict of Interest Statement

The authors declare that the research was conducted in the absence of any commercial or financial relationships that could be construed as a potential conflict of interest.
